# GTPBP4: A New Therapeutic Target Gene Promotes Tumor Progression in Non-Small Cell Lung Cancer via EMT

**DOI:** 10.1155/2022/2164897

**Published:** 2022-11-11

**Authors:** Junlu Wu, Guofei Chen, Weiwei Wang, Yang Yang, Yi Yuan, Anquan Shang, Wenqiang Quan, Lixin Wang

**Affiliations:** ^1^Department of Laboratory Medicine, Shanghai Tongji Hospital, School of Medicine, Tongji University, Shanghai 200065, China; ^2^Center for Laboratory Medicine and School of Clinical Medicine, General Hospital of Ningxia Medical University, Yinchuan 750003, Ningxia, China; ^3^Department of Laboratory Medicine, Suzhou Hospital of Integrated Traditional Chinese and Western Medicine, Suzhou 215101, China; ^4^Department of Pathology, Tinghu People's Hospital of Yancheng City, Yancheng 224001, Jiangsu, China; ^5^Department of Radiology, Luodian Hospital, Baoshan District, Shanghai 201908, China; ^6^Center for Laboratory Medicine, The First Affiliated Hospital of Xi'an Jiaotong University, Xi'an 710061, China

## Abstract

Lung cancer has a complex etiology involving multiple regulatory systems. Uncertainty about the biology and evolution of lung cancer has made it difficult to improve its poor prognosis. To create efficient therapeutic targets and optimal molecular screening tools for lung cancer, the most important task seems to be to understand how it develops and progresses. The expression and regulation of GTPBP4 in non-small cell lung cancer (NSCLC) are not well understood. Using methods such as knocking down GTPBP4 in lung cancer cells and establishing a mouse lung cancer model, we found that the expression of GTPBP4 was upregulated in human lung adenocarcinoma cells and tissues, and that knocking down the expression of the GTPBP4 gene in A549 and Calu-1 lung adenocarcinoma cells can inhibit the proliferation of lung adenocarcinoma cells and reduce their invasion ability. The results of the mouse lung cancer model showed that the lung weight and the number of lung surface nodules decreased significantly in the LLC-GTPBP4 KO group. The mechanism by which GTPBP4 regulation affects the progression of lung adenocarcinoma may be related to the regulation of EMT. From this study, new research ideas emerge to explore GTPBP4 as a biomarker and therapeutic target for early diagnosis and treatment of lung cancer.

## 1. Introduction

According to the World Health Organization, lung cancer still has the highest mortality rate in the world. According to recent estimates, the number of deaths from lung cancer in China will reach 1.8 million in 2020. This would significantly surpass the number of deaths from other cancers in the country and rank first in overall cancer deaths [1]. Non-small cell lung cancer (NSCLC) is the most common histologic form of lung cancer and accounts for more than 85 percent of all lung malignancies. Non-small cell lung cancer is also the most deadly. Due to the insidious symptoms and highly invasive nature of early-stage NSCLC, which is often associated with multiple metastases to lymph nodes and other organs, there is no effective early detection program, and approximately three-quarters of patients are not detected until the mid-and advanced stages of their disease [2]. Despite advances in surgery, targeted treatment, immunotherapy, radiation, and chemotherapy, non-small cell lung cancer still has a low 5 year survival rate and a dismal prognosis despite recent advances in medical science. Recurrence and metastasis are thought to be the main causes of this disease. Several variables, including genetics and the environment, contribute to the development and incidence of lung cancer. The etiology of lung cancer is complicated and involves a number of complex regulatory processes. The dismal prognosis of lung cancer has not been significantly improved due to a lack of comprehensive knowledge of the pathophysiology and development process, as well as effective prevention, early detection, and treatment strategies [3]. It appears that the most urgent task in the development of lung cancer diagnosis and therapy is to determine the mechanism of its development and progression in order to identify effective therapeutic targets and optimal molecular screening techniques.

A linear DNA sequence of 12285 bp in length for human GTP-binding protein 4 (GTPBP4), located on chromosome 10pl5-p14, was identified in GeneBank (DQ496111.1). The encoded protein is the GTP-binding protein, and the gene is encoded by the GTP-binding protein. The GTPBP4 gene in humans has a high degree of similarity to the NOG1-1 gene in yeast. The GTPBP4 provides instructions for encoding a tiny GTP-binding protein located in the nucleus. Nog1 is a protein that plays a key role in the biological functions of ribosome synthesis, particularly in the production of the ribosomal 60S subunit, which is essential for ribosome synthesis [4]. The relationship between GTPBP4 and tumor pathogenesis is currently under investigation, and various study results suggest that GTPBP4 plays different roles in different diseases. A number of recent studies have shown that GTPBP4 is associated with the cell proliferation of gastric cancer [5], the prognosis of liver cancer patients [6], and the spread of colorectal cancer [7]. It has also been reported that silencing GTPBP4 in lung cancer cell lines results in significant inhibition of cell proliferation, angiogenesis, clone formation, and tumor development in nude mice [8] although the specific processes and signaling pathways involved in NSCLC remain unclear.

As a result of specific programs, epithelial-mesenchymal transition (EMT) is a biological process in which epithelial cells undergo transformation into mesenchymal phenotypes as a result. It is crucial for embryonic development, cancer metastasis, and fibrotic diseases, among others [9, 10]. EMT leads to loss of tight junctions between epithelial cells, loss of cell polarity, and acquisition of a mesenchymal cell phenotype with high motility and invasion, which is an essential cytological basis for invasion and metastasis of malignant tumors [11]. Extrinsic signaling molecules such as transforming growth factor beta (TGF-beta), fibroblast growth factor (FGF), epidermal growth factor (EGF), PDGF-A, and PDGF-B have been shown to be involved in the EMT process [12]. Some transcription factors, known as EMT-inducible transcription factors, are the primary regulators of EMT. These transcription factors include *Snail, Slug, ZEB1/2*, and *Twist1/2* [13, 14]. EMT-inducible transcription factors promote the transcription of genes normally expressed in mesenchymal cells, such as *N-cadherin, vimentin,* and *fibronectin,* while inhibiting the expression of epithelial markers such as *E-cadherin* [15]. *N-cadherin, vimentin*, and *fibronectin* are examples of such genes. One of the most striking changes during the EMT process is the downregulation of E-cadherin expression. Inhibition of E-cadherin expression has been shown to decrease adhesion between epithelial cells, resulting in loss of cell polarity and cell migration [13, 16]. The extent of GTPBP4 expression and unique regulatory mechanisms in NSCLC has rarely been studied. Therefore, this research aims to elucidate the impact of GTPBP4 on NSCLC proliferation and metastasis and the particular mechanism by which this occurs, using in vitro and in vivo studies to discover novel therapeutic targets for NSCLC.

## 2. Materials and Methods

### 2.1. Publicly Available Databases Analysis

The Cancer Genome Atlas (TCGA) database (https://portal.gdc.cancer.gov/) was used to collect gene expression profiles for NSCLC patients.

### 2.2. General Information of Lung Cancer Tissue Microarray

A lung cancer tissue microarray (ZL-LUC601) was bought from Shanghai Zhuoli Biotechnology Co., Ltd. The clinical pathological data comprised gender, age, pathological grade, clinical stage, and survival duration.

### 2.3. Cell Culture and Cell Transfection

Human NSCLC cell lines H838, H2347, Calu-1, A549, human embryonic lung fibroblast MRC-5, and mouse Lewis lung cancer cells LLC were obtained from the Chinese Academy of Sciences Cell Bank. Cells were grown in DEME (GIBCO, Grand Island, NY, USA) medium with 10% fetal bovine serum and 5% CO_2_ at 37°C. A549 and LLC cells were seeded into 6-well plates one day before transfection and achieved 70% confluence the next day. These shRNAs were made by Shanghai Genechem Co.,Ltd. In a 6-well plate, cells at a density of 2.0 × 10^5^ cells per well were transfected with shRNA using Lipofectamine 2000 (Invitrogen, Carlsbad, CA, USA). After 6 h, the medium was changed with FBS. Follow-up tests were done 24 h following transfection. The transfection experiments were carried out as directed by the reagent, with blank and negative controls set up simultaneously.

### 2.4. Western Blotting Analysis

Western blotting analysis was performed as described [17]. Briefly, Frozen mouse lung tissue or NSCLC cells were homogenized on ice in 5 × radioimmunoprecipitation assay buffer (RIPA buffer; 50 mM Tris [pH 8.0], 150 mM NaCl, 0.1% SDS, 0.5% sodiumdeoxy-cholate, 1% NP-40, and 5 mM EDTA) supplemented with protease inhibitor cocktail (Sigma-Aldrich, St.Louis, MO, USA), followed by centrifugation at 16,000 × g for 30 minutes at 4°C to collect the supernatants. 30 *μ*g total protein were loaded on 10% SDS (Sigma-Aldrich, St.Louis, MO, USA) polyacrylamide gels, electrophoresed and blotted onto Hybond-C Extra membranes (Amersham Bioscience, Buckinghamshire, UK). Primary antibodies used for western blot analysis included the following: rabbit anti-E-Cadherin (clone: 24E10, CST, Danvers, MA, USA), rabbit anti-Vimentin (clone: D21H3, CST, Danvers, MA, USA), rabbit anti-GTPBP4 (ab184124, Abcam, Cambridge, MA, USA), mouse anti-*β*-actin (sc-8432, Santa Cruz Biotechnology, Santa Cruz, CA, USA), HRP-conjugated goat anti-rabbit (sc-2004, Santa Cruz Biotechnology, Santa Cruz, CA, USA), or rabbit anti-mouse (P0260, Dako Cytomation, Glostrup, Denmark) was used as a secondary antibody. Lightning ECL substrate (CAT:^#^12630, CST, Danvers, MA, USA) was developed. According to the manufacturer's instructions substrate solution A and substrate solution B were mixed in a 1 : 1 ratio and distributed evenly on the membrane. After 2 minutes, the substrate mixture was removed and the chemiluminescence was detected using Chemiluminescence Image Analysis System (Tanon, Shanghai, China). Densitometric analyzes were performed with the image processing program Image J (NIH, Version 1.43).

### 2.5. Immunohistochemistry Staining

The lung cancer tissue microarray was placed in an oven at 60°C for 30 min, deparaffinized with xylene and ethanol with gradient concentration, then put into water, washed with PBS, and then repaired with 0.01 mol/L citric acid for 15 min at 98°C. The lung tissue chips were washed with PBS and then washed with 3% H_2_O_2_; incubated at room temperature to remove endogenous peroxidase activity; incubated with primary antibody GTPBP4 (1 : 100) overnight at 4°C, and secondary antibody incubation and staining were carried out on the second day according to the instructions of the EnVision™+ System/HRP kit (K400711-2, Dako Cytomation, Glostrup, Denmark). After incubation of secondary antibodies, cells were washed 3 times with PBS. Hematoxylin red staining, ethyl alcohol differentiation, gradient dehydration, xylene lucency, and neutral rubber sealing film were used. The positive expression product of GTPBP4 protein is yellow-brown, and we further refer to the Fromowitz semiquantitative integration method [18] to score the results, briefly 0 points for no coloration, 1 point for light yellow, 2 points for brownish yellow, and 3 points for tan. Positive range: <5%, 0 points; 5% to 25%, 1 point; 26% to 50%, 2 points; 51% to 75%, 3 points; and >75%, 4 points. The addition of the two results <2 points is considered negative (−), 2-3 points is weakly positive (+), 4-5 points is moderately positive (++), and 6-7 points is strongly positive (+++). The staining intensity score×staining positive rate score was the total score; the total score ≤ 3 was defined as low expression, and a total score > 3 was defined as high expression

### 2.6. Cell Counting Kit-8 (CCK-8) Assay

A549 (blank control), A549 sh-NC(negative control) group, and A549-sh-GTPBP4 cells were seeded in a 96-well plate (6 × 10^3^ cells/well) and cultured in an incubator 5% CO_2_ at 37°C. After 6 hours, 10 *μ*l of CCK-8 solution was added, incubated under normal culture conditions for 2 hours, and the absorbance at 450 nm was measured with a microplate reader; the same operation was performed on the second day until the sixth day; the measured absorbance at each time point was input into GraphPad Prism 8.0 software for plotting and statistical analysis.

### 2.7. Cell Invasion Assay (Transwell)

Take out the prefabricated Transwell chamber (Corning, NY, USA) with matrigel from the −20°C refrigerator, incubate at 37°C incubator for 2 h, adjust the cell concentration to 5 × 10^4^ cells/ml, pipette 500 *μ*l of the cell suspension, and gently add it to the upper layer of the transwell chamber. The same volume of serum-containing medium was added to the lower chamber and cultured in a 37°C, 5% CO_2_ incubator for 24 h. The upper chamber of the Transwell was taken out and wiped to remove non-invasive cells on the upper layer of the membrane. After methanol fixation and crystal violet staining, 4–6 visual fields under the microscope were randomly selected to count the number of stained cells.

### 2.8. Construction of Mouse Lung Cancer Model by Tail Vein Injection

Twenty 4-week-old female C57BL/6 mice were housed in the SPF animal room of Tongji Hospital Affiliated to Tongji University. The mice were randomly divided into 2 groups, 10 mice in each group, and were injected into the tail vein. Method is as previously reported [19]. Cell preparation: LLC cells and LLC-GTPBP4 ko cells were digested 1 h before the tail vein injection of mice, centrifuged to wash the cells twice with serum-free medium DMEM, and the cell concentration of the two groups was adjusted to 1 × 10^6^ cells/ml. The mouse was fixed in the mouse tube and placed on the mouse tail injection vein imaging device, and the infrared light source was turned on to illuminate the tail of the mouse to find and select the vein for injection. After sterilizing the mouse tail, used a sterile syringe (1 ml, 25G) for tail vein injection. The number of cells injected per mouse was 1 × 10^5^ cells, and the volume is 100 *μ*l. Observation and lung harvesting: the state of the mice was observed every 3 days. On the 21^st^ day, mice were euthanized, and lungs were lavaged, removed, weighed, and fixed for histological examination. Lung tumor nodules were carefully counted under a dissecting microscope as previously described [20]. The lung tissues were frozen at −80°C or stored in neutral formalin at 4°C.

### 2.9. Statistical Analysis

Each experiment was performed independently ≥ 3 times. Results are presented as mean ± standard deviation (SD). All data were analyzed using Graphpad Prism8.0 (San Diego, CA). The receiver operating characteristic (ROC) curve was used to determine the sensitivity and specificity of the index, and the area under the curve (AUC) was used to assess the diagnostic value of the test. A Student's *t*-test was used to analyze differences between groups, and *P* < 0.05 was considered to indicate a statistically significant difference.

## 3. Results

### 3.1. The Expression and Diagnostic Value of GTPBP4 in Lung Cancer

**Figure 1:**
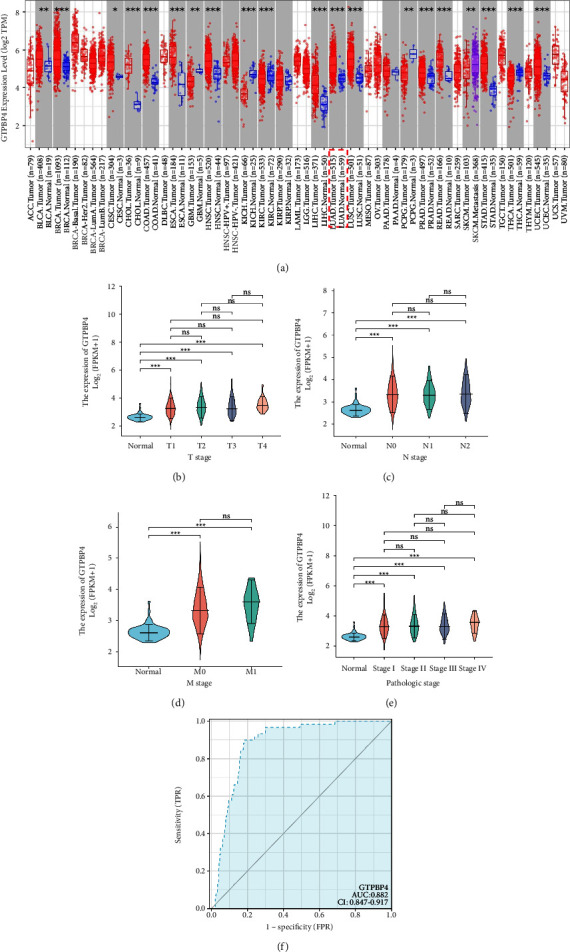
The expression and diagnostic value of GTPBP4 in lung cancer. (a) GTPBP4 was upregulated in the majority of human cancer types available via TCGA, including LUAD. (b–e) GTPBP4 expression was increased in primary tumor LUAD samples compared to normal samples but has no statistically significant difference in T stage, lymph node metastasis status (N), distant metastasis status (M), and tumor pathological stage of lung cancer.(N0: no regional lymph node metastasis; N1: metastases in 1–3 axillary lymph nodes; N2: metastases in 4–9 axillary lymph nodes; M0: no distant metastasis; M1: distant metastasis). (f) The diagnostic value of GTPBP4 in lung cancer (^*∗∗*^*P* < 0.01; ^*∗∗∗*^*P* < 0.001).

To examine the expression of GTPBP4 in lung cancer, we assessed the published data for cancer and normal tissues from the TCGA databases. The results showed a significant upregulation of GTPBP4 in several human cancer subtypes (*P* < 0.05), including LUAD compared with noncancerous lung tissues in TCGA (Figure 1(a)). GTPBP4 expression was higher in LUAD primary tumor tissues than in adjacent normal tissues. However, the effects of T stage, lymph node metastasis status (N), distant metastasis status (M), and tumor pathological stage of lung cancer did not lead to differential expression of GTPBP4 in lung cancer (Figures 1(b)–1(e)). GTPBP4 also had a good value in the diagnosis of lung cancer; the area under the receiver operating characteristic curve (AUC) was 0.882, indicating that GTPBP4 might be a good biomarker for the diagnosis of lung cancer (Figure 1(f)).

### 3.2. GTPBP4 Protein Expression Was Significantly Increased in NSCLC

**Figure 2:**
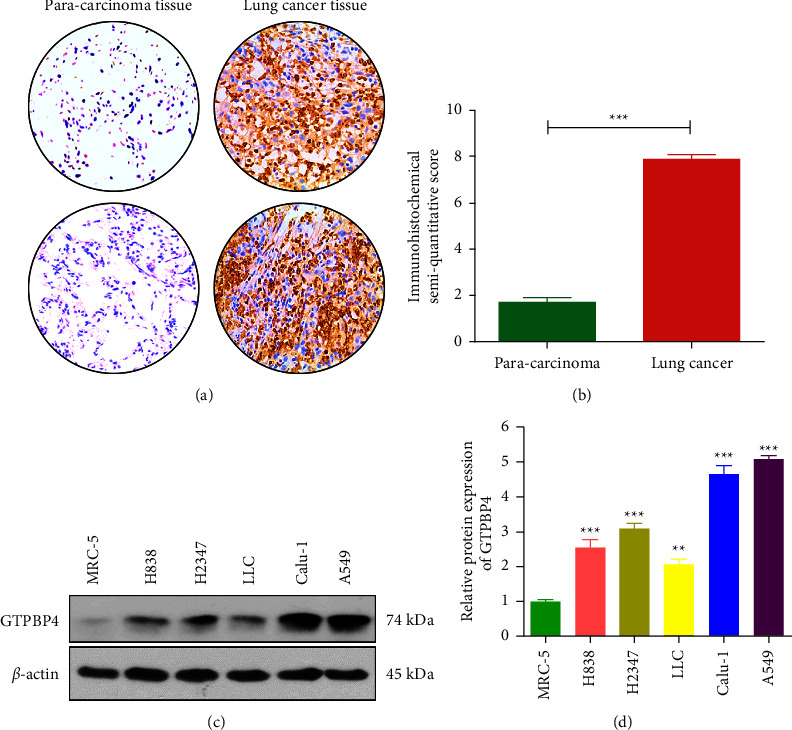
GTPBP4 protein expression was significantly increased in NSCLC. (a) The expression of GTPBP4 in lung adenocarcinoma tissue microarray was detected by immunohistochemistry (400✕). (b) Fromowitz semiquantitative integration method showed that the expression of GTPBP4 in lung cancer tissues was significantly higher than that in adjacent tissues (^*∗∗∗*^*P* < 0.001). (c-d) Western blotting showed that GTPBP4 was highly expressed in lung adenocarcinoma cell lines compared with the control group (normal human embryonic lung cell MRC-5), and the difference was statistically significant (*n* = 4 per group, ^*∗∗*^*P* < 0.01; ^*∗∗∗*^*P* < 0.001).

We then performed immunohistochemical staining on tissue chips containing 32 NSCLC cancer tissues and paracancerous tissues. Our results showed that GTPBP4 protein was highly expressed in NSCLC tissues and lowly expressed in the corresponding paracancerous tissues of NSCLC; GTPBP4 highly expressed in lung cancer cells were expressed in the cytoplasm and nucleus (Figure 2(a)); the difference between the two groups was statistically significant (*t* = 5.214; *P*=0.019; Figure 2(b)). We further detected and analyzed the expression of GTPBP4 protein in human NSCLC cell lines H838, H2347, Calu-1, and A549 and mouse lung cancer cells LLC by immunoblotting. The results showed that GTPBP4 was highly expressed in lung adenocarcinoma cell lines compared with the control group (normal human embryonic lung cell MRC-5), and GTPBP4 was the highest expression in A549 cells (Figure 2(c)). The gray value of protein bands was analyzed by Image J, and the difference was statistically significant (*n* = 4 per group; *P* < 0.05; Figure 2(d)).

### 3.3. Knockdown of GTPBP4 Suppressed A549 and Calu-1 Cells Proliferation

**Figure 3:**
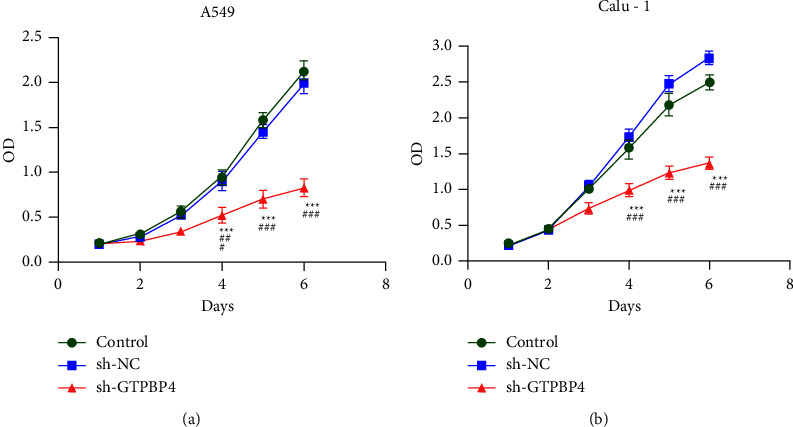
Knockdown of GTPBP4 suppressed A549 and Calu-1 Cells proliferation. CCK8 assays showed that the proliferation of sh-GTPBP4 group A549 and Calu-1 cells was significantly inhibited when the cells were transfected after 6 days, compared with sh-NC (negative control) group and control (blank control) group (both *n* = 3 per group, ^*∗∗∗*^*P* < 0.001,^###^*P* < 0.001).

Based on the results of western blotting, A549 and Calu-1 cells line were selected for using in subsequent experiments. CCK8 assays showed that RNA interference of GTPBP4 in A549 and Calu-1 cells significantly decreased the absorbance value of the sh-GTPBP4 group when the cells were transfected after 6 days, compared with sh-NC (negative control) group and control (blank control) group (both *n* = 3 per group, *P* < 0.001; Figures 3(a)–3(b)). The difference between the two control groups was not statistically significant (*n* = 3 per group, *P* > 0.05; Figures 3(a)–3(b)).

### 3.4. Knockdown of GTPBP4 Inhibited the Invasive Ability of A549 and Calu-1 Cells

**Figure 4:**
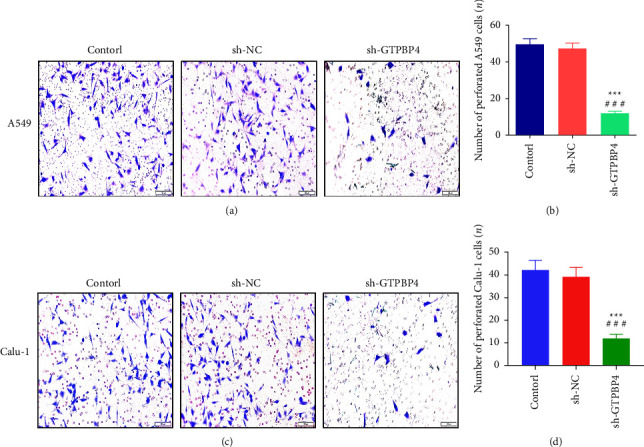
Knockdown of GTPBP4 inhibited the invasive ability of A549 and Calu-1 cells. (a-b) The number of transmembrane cells in the sh-GTPBP4 group of A549 cells was significantly decreased compared with the control group, and the difference was statistically significant *n* = 3 per group, ^*∗∗∗*^*P* < 0.001, ^###^(*P* < 0.001); (c−d). The number of transmembrane cells in the sh−GTPBP4 group of Calu^−1^ cells was significantly decreased compared with the control group, and the difference was statistically significant (*n* = 3 per group, ^*∗∗∗*^*P* < 0.001, ^###^*P* < 0.001).

We next used transwell cell invasion assay in vitro to verify the effect of RNA interference of GTPBP4 in A549 and Calu-1 cells. The results showed that the number of transmembrane cells in control group, sh-NC group, and sh-GTPBP4 group in A549 cells was (49.70 ± 9.73), (47.40 ± 9.06), and (12.00 ± 3.13), respectively. Compared with the control group, the number of transmembrane cells in the GTPBP4-shRNA group in A549 cells was significantly decreased, and the difference was statistically significant (*n* = 6 per group, both *P* < 0.001; Figures 4(a)–4(b)).

Moreover, the number of transmembrane cells in control group, sh-NC group, and sh-GTPBP4 group in Calu-1 cells was (42.33 ± 10.29), (39.17 ± 10.25), and (11.83 ± 4.36), respectively. Compared with the control group, the number of transmembrane cells in the GTPBP4-shRNA group in Calu-1 cells was significantly decreased, and the difference was statistically significant (*n* = 6 per group, both *P* < 0.001; Figures 4(c)–4(d)).

### 3.5. Knockout of GTPBP4 Inhibited NSCLC Proliferation in Mice

**Figure 5:**
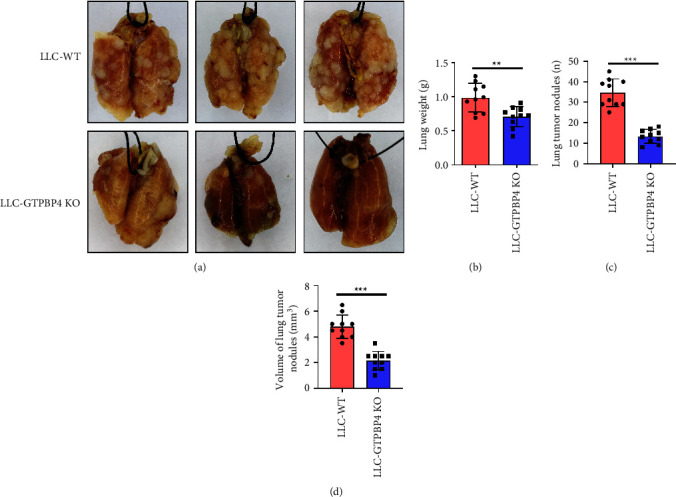
Knockout of GTPBP4 inhibited NSCLC proliferation in mice. (a) A mouse lung cancer model established by tail vein injection. At 21 days, the complete morphology of the lung tissue removed after the mice were sacrificed. (b) The lung weight of LLC-GTPBP4 KO mice was significantly reduced (*n* = 10 per group, ^*∗∗*^*P* < 0.01). (c) The number of lung tumor nodules in the LLC-GTPBP4 KO group was significantly less than that in the LLC-WT group (*n* = 10 per group, ^*∗∗∗*^*P* < 0.001). (d) The volume of lung tumor nodules in the LLC-GTPBP4 KO group was significantly smaller than that in the LLC-WT group (*n* = 10 per group, ^*∗∗∗*^*P* < 0.001).

In order to further verify the results of in vitro experiments, we used tail vein injection of LLC-WT and LLC-GTPBP4 KO cells to establish a mouse lung cancer model in C57BL/6 mice and observed the effect of GTPBP4 protein on the development of NSCLC. On the 21^st^ day after the tail vein injection, the mice were uniformly dissected, and the lung weight, the number of lung tumor nodules, and the diameter of tumor nodules were evaluated. Our results showed that the lung weight of LLC-GTPBP4 KO mice was significantly reduced (*n* = 10 per group, *P* < 0.01; Figures 5(a)–5(b)), and the number of lung tumor nodules in the LLC-GTPBP4 KO group was significantly less than that in the LLC-WT group, and the difference was statistically significant (*n* = 10 per group, *P* < 0.001, Figure 5(c)). The volume of lung tumor nodules in the LLC-GTPBP4 KO group was significantly smaller than that in the LLC-WT group (*n* = 10 per group, *P* < 0.001, Figure 5(d)). The results of vivo experiments indicated that GTPBP4 promoted the proliferation of lung tumors in mice.

### 3.6. GTPBP4 Promoted NSCLC Proliferation by Regulating EMT

**Figure 6:**
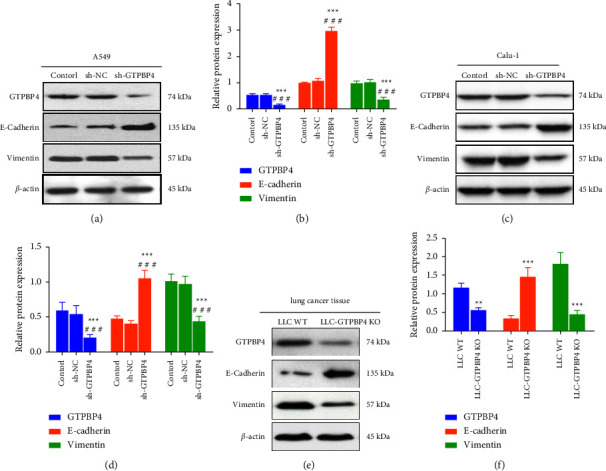
GTPBP4 promoted NSCLC proliferation by regulating EMT. (a-b) The expression of E-cadherin protein was significantly increased, but the expression of Vimentin was significantly decreased in A549-sh-GTPBP4 group compared with the two control groups (*n* = 3 per group, ^*∗∗∗*^*P* < 0.001, ^###^*P* < 0.001). (c-d) The expression of E-cadherin protein was significantly increased, but the expression of Vimentin was significantly decreased in Calu−1-sh-GTPBP4 group compared with the two control groups *n* = 3 per group, ^*∗∗∗*^*P* < 0.001, ^###^(*P* < 0.001). (e-f) The expression of E-cadherin protein was also significantly increased and the Vimentin protein was significantly decreased in the LLC-GTPBP4 KO group compared with the LLC-WT group in mouse lung cancer tissue (*n* = 3 per group, ^*∗∗*^*P* < 0.01; ^*∗∗∗*^*P* < 0.001).

EMT process was associated with migration, invasion, and drug resistance of lung cancer. To investigate whether GTPBP4 promoted NSCLC proliferation by regulating EMT, we examined the effect of knockdown of GTPBP4 on EMT-related proteins in NSCLC cell line A549, Calu-1, and mouse lung cancer tissues. Our results showed that the expression of E-cadherin protein, a marker of epithelial cells, was significantly increased in A549-sh-GTPBP4 group compared with the two control groups while the expression of protein of mesenchymal cell marker Vimentin was significantly decreased (*n* = 3 per group, *P* < 0.001; Figures 6(a)–6(b)). And, we found that the same trend of results was also obtained in Calu-1 cells (*n* = 3 per group, *P* < 0.001; Figures 6(c)–6(d)). Meanwhile, the expression of E-cadherin protein in LLC-GTPBP4 KO group was also significantly increased in mouse lung cancer tissue, the Vimentin protein was significantly decreased in the LLC-GTPBP4 KO group compared with the LLC-WT group, and the difference was statistically significant (*n* = 3 per group, *P* < 0.01; Figures 6(e)–6(f)).

## 4. Discussion

Lung cancer is one of the most prevalent malignant tumors in the world, and it is also the biggest cause of cancer-related mortality, accounting for 18.4% of all cancer-related deaths [21]. NSCLC is the most common histological subtype of non-small cell lung cancer [3] and has gradually become a major challenge to human health. In spite of significant advancements in cancer therapy, the prognosis for individuals with NSCLC remains dismal, with survival rates below 20% [22]. It is thus critical to conduct thorough study and research to identify novel therapeutic targets for NSCLC. GTPBP4 is a recently found GTPase that is a member of the GTPBP family and is a functional protein of significant importance [5]. In recent investigations, it has been discovered to be strongly associated with the onset, development, and prognostic monitoring of some malignancies, which has sparked broad interest and has the potential to be employed as a cancer therapeutic target. As a result of our research, we discovered that the expression of the GTPBP4 gene was upregulated in both human NSCLC tissues and cells; we also discovered that knocking out the expression of the GTPBP4 gene in lung cancer cells could inhibit the proliferation of lung cancer cells, reduce their invasive ability, and inhibit the growth of lung cancer in mice; the mechanism may be that GTPBP4 regulates EMT, which could slow the progression of NSCLC.

At the moment, the status of research into the GTPBP4 gene, particularly its association to tumors, is uncertain. Li et al. [5] studied the interaction between GTPBP4 and p53 in gastric cancer and promoted gastric cancer progression by regulating downstream negative effectors. Yu et al. [7] found that GTPBP4 specifically induced filamentous actin rearrangement by inhibiting RhoA activity and promoted colorectal cancer metastasis. Knockdown of GTPBP4 gene in hepatocellular carcinoma (HCC) inhibited cell proliferation, impaired colony formation ability, induced cell cycle arrest in G2/M period, and promoted apoptosis in HCC cell lines. Besides, in vivo xenograft nude mice model revealed that GTPBP4 knockdown could significantly suppress HCC tumorigenesis [8]. Recently, studies reported that in glioma cell lines, GTPBP4 inhibited cell proliferation by regulating Merlin protein and CyclinD1 protein [23]. Based on our previous research, we discovered that the expression levels of GTPBP4 in different malignant tumors were significantly different, and that the effect was two-sided, suggesting that GTPBP4 has two distinct roles in different tumors, one as a tumor suppressor and the other as a tumor promoter. So far, investigations on GTPBP4 in lung cancer, particularly non-small cell lung cancer (NSCLC), have been published very seldom.

As part of this study, we first looked at the TCGA database and discovered that the expression of GTPBT4 was significantly increased in lung cancer. We then looked at lung tissue microarray immunohistochemical staining and discovered that the expression level of GTPBP4 in NSCLC tissue was significantly higher than the expression level in the adjacent control group. According to our findings, human NSCLC cell lines H838, H2347, Calu-1, and A549 had considerably higher levels of GTPBP4 protein expression than normal, with the expression of GTPBP4 protein increasing the most strongly in A549 cells. As a result, the A549 cell line was chosen as the research subject for the remainder of this investigation. Using shRNA-GTPBP4 transfect A549 cells to target the expression of GTPBP4 gene, we discovered that silencing GTPBP4 may dramatically decrease the proliferation of A549 cells, as well as the capacity of these cells to invade other cell types (invasive ability). In addition, we developed a mouse lung cancer model [24] in order to better investigate the significance of GTPBP4. The weight of lung tumors in mice was dramatically decreased by tail vein injection of GTPBP4ko-LLC cells, and the number of lung surface nodules was also significantly reduced when compared with the control group, demonstrating that GTPBP4 deletion may prevent the formation of lung tumors in mice. Using the aforementioned in vitro and in vivo experimental data, we discovered that GTPBP4 can accelerate the advancement of non-small cell lung cancer via controlling the growth, proliferation, and invasion of lung cancer cells.

Previous research has shown that the EMT process is related with lung cancer migration, invasion, and treatment resistance [25]. To investigate whether the expression level of GTPBP4 is connected to the EMT state of lung cancer cells, we conducted a preliminary investigation in which we looked at the expression level of GTPBP4. Following our findings, we discovered that interfering with the expression of GTPBP4 in A549 cells resulted in a significant increase in the expression of EMT-relatedE-cadherin protein in A549 cells, while the expression of Vimentin protein was significantly decreased; the same findings were also discovered in lung cancer tissues. These findings imply that interfering with GTPBP4 alters the EMT state of lung cancer cells, and that GTPBP4 may enhance the proliferation of lung cancer cells through controlling the EMT process. Although our findings in this research demonstrated that GTPBP4 and EMT have an influence on one another, the link between GTPBP4's upstream and downstream regulators has not yet been determined. Therefore, more in vitro and in vivo investigations should be carried out in order to better understand the link and molecular mechanism of GTPBP4 and NSCLC in greater depth.

In conclusion, our findings indicate that the expression of GTPBP4 was upregulated in both human NSCLC tissues and cells, that silencing the expression of GTPBP4 gene in lung cancer cells inhibited the proliferation of lung cancer cells and reduced their invasive ability, and that the mechanism may involve regulating EMT to affect lung cancer progression. This study laid the groundwork for future research into the role of GTPBP4 in the occurrence and progression of lung cancer, and it generated new research ideas for investigating whether GTPBP4 can be used as a biomarker and therapeutic target for the detection and treatment of lung cancer in its early stages.

## Data Availability

The data used to support the findings of this study are included within the article. Further inquiries can be directed to the corresponding authors.

## Abbreviations

NSCLC: Non-small cell lung cancer
GTPBP4: GTP-binding protein 4
EMT: Epithelial-mesenchymal transition
TGF-*β*: Transforming growth factor beta
FGF: Fibroblast growth factor
EGF: Epidermal growth factor.
